# Supplementary material for: A Sweet Cherry Glutathione S-Transferase Gene, PavGST1, Plays a Central Role in Fruit Skin Coloration
Source: Cells. 2022 Mar 30;11(7):1170. doi: 10.3390/cells11071170 (PMC8997526; doi:10.3390/cells11071170)
Supplement: Supplementary file 1 [file cells-11-01170-s001.zip › cells-1622302-supplementary.pdf]

Table S1 *Cis*-acting elements found in the upstream region of *PavGST1* gene

Motif	sequence	Position	Strand	function
TATA-box	ATTATA	62	+	core promoter element around -30 of transcription start
	ATATATATAT	77	+	
	ccTATAAAaa	140	+	
	TATTTAAA	976	+	
CAAT-box	CAAT	52, 247	+	common cis-acting element in promoter and enhancer regions
	TGCCAAC	213	+	
	CAAAT	750	+	
	CAAAT	866, 945	-	
TCT-motif	TCTTAC	660	+	part of a light responsive element
ABRE	ACGTG	398, 497, 605	+	cis-acting element involved in the abscisic acid responsiveness
TC-rich repeats	GTTTTCTTAC	656	+	cis-acting element involved in defense and stress responsiveness
	ATTCTCTAAC	1686	-	
G-box	CACGTC	496	-	cis-acting regulatory element involved in light responsiveness
MYB	CAACCA	712	+	
	CAACCA	1085	+	
	CAACCA	1402	+	
TGACG-motif	TGACG	709	-	cis-acting regulatory element involved in the MeJA-responsiveness
LTR	CCGAAA	261	-	cis-acting element involved in low-temperature responsiveness
TCCC-motif	TCTCCCT	1305	+	part of a light responsive element
G-Box	CACGTT	397, 604	-	cis-acting regulatory element involved in light responsiveness
GATA-motif	GATAGGA	139, 1452	-	part of a light responsive element
	GATAGGA	506	+	part of a light responsive element
CGTCA-motif	CGTCA	709	+	cis-acting regulatory element involved in the MeJA-responsiveness

Table S2: Primers used in this study.

Primer name	Sequences (5′→3′)
PavGST1-RNAi-F	AGTAAGGTTACCGAATTC ACCTCAATTCCTCTCCCGTCAG
PavGST1-RNAi-R	GAGCTCGGTACCGGTACCTGCCCCAATTTAGCTTCATCAA
PavGST1-ox-F	GGGGACTCTTGACCATGGATGGTTGTGAAAGTGTATGGTC
PavGST1-ox-R	AAATTCGAGCTGGTCACCCTAGTAGTCACTAGCAAGGCTCA
PavGST1-F	ATGGTTGTGAAAGTGTATGGTC
PavGST1-R	CTAGTAGTCACTAGCAAGGCTCA
PavMYB10.1-F	ATGGAGGGCTATAACTTGGGTGTGAG
PavMYB10.1-R	TTAGTCCTTCTGAACATTGGTACAC
PavMYB75-F	ATGGAGGGAAATAACTTGGATGTG
PavMYB75-R	TTATTCTTCTTTTGAATGATTCC
PavbHLH-F	ATGCATCTCCAGAGTGCTCGTATAC
PavbHLH-R	CTAGGAATCAGATTGGGGAATTATTT
PavMYB10.1-q-F	GTTCCTTACAAAGCAGGGTTGAA
PavMYB10.1-q-R	GCTTTGGGGTTGAGGTCTTATTA
PavMYB75-q-F	GAGCTGTAGACTAAGGTGGTTGA
PavMYB75-q-R	CACCAGAGAATTCGTCCGCAGT
Pavactin-F	GCTGGTCGTGACCTCACAGAT
Pavactin-R	CCAATTGTGATTACTTGGCCA
PavbHLH-q-F	GACGAGGAGGCAGAGTCAGA
PavbHLH-q-R	AGTCAGCTTGGCGCTGCTGA

PavWD40-q-F	CAGTAAGACCAGCGAGTTCTG
PavWD40-q-R	AATCGAAGATCCTCACGGACC
PavPAL-q-F	TGAGATTGCCATGGCATCTTATTG
PavPAL-q-R	TGTGTTCCTCAAGTTCTCCTCC
PavC4H-q-F	AGCCACATGACCACGTAACCTAC
PavC4H-q-R	TCATGATTGCGACGATGATGGCG
Pav4CL-q-F	CAGTTCGTCGACAAGCTTCGA
Pav4CL-q-R	TCCTTCTCGTTGGCCTCCGAG
PavCHS-q-F	CAACTATGTGTGAGTACATGGCA
PavCHS-q-R	TTGTTGGTACATCATGAGGCGC
PavCHI-q-F	CCGTTGAGTTCTTCAGAGAGAT
PavCHI-q-R	GATAACCTCGCAGCTACACTCTG
PavF3H-q-F	GCCAGAGGGATGGAGAGAGGT
PavF3H-q-R	TCAGTGTGGCGCTTCAGGCC
PavFLS-q-F	GCCTCTTGACTCTGACTCTAT
PavFLS-q-R	CTGAGCAGCTTCTCCACCAC
PavDFR-q-F	CACTGTTCGAGCCACCGTGCGA
PavDFR-q-R	TATGTGAGAGAGGCAGAGGTCG
PavANS-q-F	CGAACAGGAGAAGAGCACTGAT
PavANS-q-R	AGAGGCCTGGTCATTGGCATAC
PavLAR-q-F	CTGCTAAGAGAGCATGAGATTGA
PavLAR-q-R	AACGGTGGAAGAACCTCAGAAGG

PavANR-q-F	CATATATGTCAGCACTGGTGACT
PavANR-q-R	CTAATCAGCAGATCAACATC
PavUFGT-q-F	GTCTCACCTGCTTGATCACCGA
PavUFGT-q-R	TGTTGGCATTCTTGGTGATGAG

Supplementary Figure S1

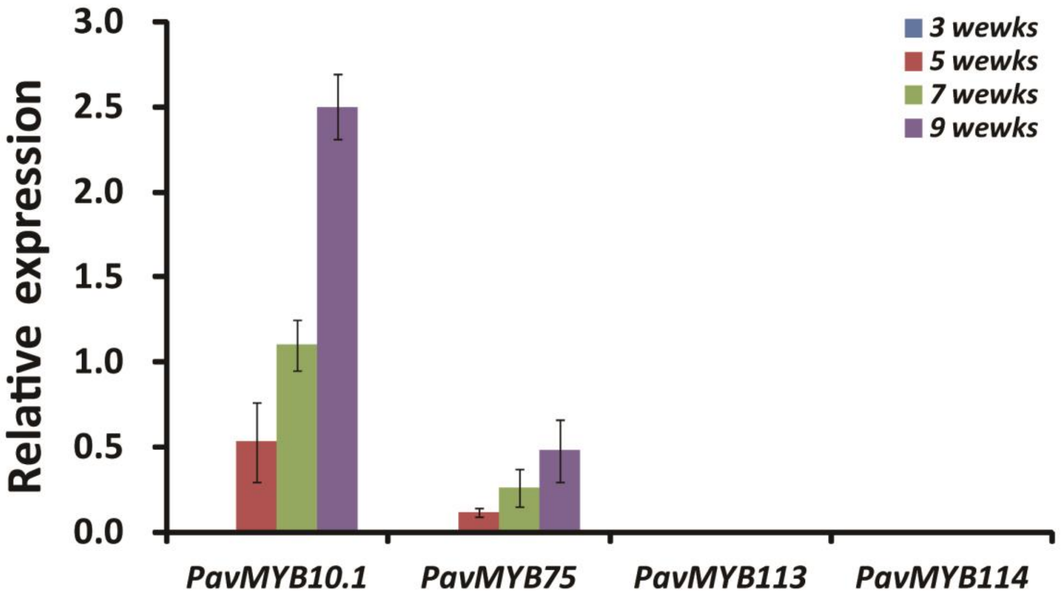

Figure S1. Expression profiles of anthocyanin-related R2R3-MYB TF family members during sweet cherry fruit growth and development. Expression profiles of Arabidopsis homologous anthocyanin-related R2R3-MYB TF family members PavMYB75/PAP1 (Pav_sc0000464.1_g100), PavMYB10.1 (Pav_sc0000464.1_g130), PavMYB113 (Pav_sc0000464.1_g250), and PavMYB114 (Pav_sc0000464.1_g210) during sweet cherry fruit growth and development. The sweet cherry Histone2 gene (Pav_sc0000671.1_g260.1.mk) was used as an internal control to normalize the expression of R2R3-MYB TF genes. Values represent means ± SD from three independent replicates.
